# The incidence of deltoid tear among patients with full-thickness rotator cuff tear

**DOI:** 10.1016/j.amsu.2022.104621

**Published:** 2022-09-11

**Authors:** Abdulrahman Alharbi, Mohammed J. Alsaadi, Abdulrahman M. Alfuraih, Mamdouh S. Almalki, Salem Bauones

**Affiliations:** a Medical Imaging Department, King Abdulaziz Medical City, Riyadh, Saudi Arabia; b Radiology and Medical Imaging Department, College of Applied Medical Sciences in Al-Kharj, Prince Sattam Bin Abdulaziz University, Al-Kharj, 11942, Saudi Arabia; c Radiology and Medical Imaging Department, College of Medicine, King Saud University, Riyadh, Saudi Arabia; d Medical Imaging Administration, Musculoskeletal Imaging Department, King Fahad Medical City, Riyadh, Saudi Arabia

**Keywords:** Deltoid muscle, Injury, MRI shoulder, Rotator cuff, Tears

## Abstract

**Backgroun:**

Full-thickness rotator cuff tear is common in the older population. The incidence of traumatic deltoid tears post-surgery is well addressed. However, non-traumatic spontaneous injury is not well recognized despite a few case reports and previous studies. The aim of the study is to determine the incidence and association of deltoid tear among patients with non-traumatic full-thickness rotator cuff tear using shoulder magnetic resonance imaging.

**Methods:**

A retrospective cross-sectional study was conducted of 271 shoulders magnetic resonance imaging examinations with full-thickness rotator cuff tear between 2012 and 2022. The analyzed variables were full-thickness rotator cuff tear size, tear grading (small, medium, large, and massive), muscle fatty degeneration, and deltoid tear. Acromio-humeral interval was also recorded and analyzed on the anteroposterior projection of shoulder radiographs.

**Results:**

The incidence of deltoid tear was 7% (19 cases), encountered in eleven females (6.4%) and eight males (8%) with a mean age of 65 years. Deltoid tears were located on the right side in fifteen patients (9.4%) and on the left side in four patients (3.6%). The Man-Whitney *U* test indicated a significant association between deltoid tears and full-thickness rotator cuff tear, P < 0.001. The deltoid tear was more notably associated with large and massive full-thickness rotator cuff tear (16.7% and 42.3%, respectively), P < 0.001. Acromio-humeral interval showed a significant difference between the deltoid and non-deltoid cases, P = 0.045.

**Conclusion:**

The incidence and association of deltoid tears with full-thickness rotator cuff tear with no prior surgical intervention or traumatic insults were considered significant, with a positive impact of large and massive tear size and association of muscle fatty degeneration. This association is statistically significant and should be adequately evaluated by the radiologist.

## Highlights

• Full-thickness rotator cuff tear is common in the older population.
• Non-traumatic spontaneous injury is not well addressed in the literature.
• The incidence of non-traumatic spontaneous injury of deltoid muscle reached almost 7%.
• There is a strong association between deltoid tears and the size of rotator cuff tears and tendon retraction.
• The middle segment of the deltoid muscle was the most common location of the tears.

## List of abbreviations

FT-RCT: Full-Thickness Rotator Cuff Tear
RCT: Rotator Cuff Tear
AH: acromio-humeral
PACS: picture archiving and communication system

## 1 Introduction

Full-Thickness Rotator Cuff Tear (FT-RCT) is commonly seen in older population. The etiology is either traumatic or degenerative. 36–50% of the RCT progress gradually in size, and larger tears could extend and negatively impact the surrounding structures [[1], [2], [3]]. Spontaneous deltoid injury secondary to chronic RCT is uncommonly encountered compared to post-operative deltoid damage, which is a recognized complication of shoulder surgery and acromioplasty [4]

The etiology of RCT is well described in the literature. It is addressed to variable extrinsic and intrinsic factors that repeatedly micro-traumatize the rotator cuff tendons, leading to tendinopathy or tear. Extrinsic factor comprises compression or friction by other shoulder structures over the rotator cuff tendons. This could lead to injury of the superficial fibers of the superior rotator cuff tendons [5]. The intrinsic factors include age-related degeneration, inflammation, vascular changes, and oxidative stress. The most widely accepted theory regarding the RCT is degenerative microtrauma [6].

Additionally, tears can be classified into stages based on age as described in the literature. The presence of edema and hemorrhage is considered stage 1. This is usually observed in patients under 25 years old. Stage 2 is characterized by fibrosis and tendonitis in patients between 24 and 40 years. Patients above 40 will develop tendon rupture and bone spurs, representing stage 3 [7,8].

The association of deltoid tear with RCT in patients without previous surgical repairs has rarely been reported in the literature [[9], [10], [11], [12], [13]]. There are few studies describing the imaging findings of spontaneous deltoid tear in patients with chronic FT-RCT [[14], [15], [16]]. Ilaslan et al. [15] reported 24 deltoid tears cases in patients with chronic FT-RCT, with an incidence rate of 0.3%. Those case series suggested an increase in the incidence of deltoid tear with FT-RCT.

The primary objective of this study is to evaluate the incidence of deltoid tear in patients with FT-RCT using MRI examination. The secondary objective is to assess the correlation between deltoid tear, FT-RCT size and grading, muscle fatty degeneration, and acromio-humeral (AH) interval. This study will provide more insight to deltoid tear in a patient with FT-RCT. Furthermore, it will enrich the literature about the deltoid tear and FT-RCT association to increase the attention of reporting radiologists and treating orthopaedics to deltoid tears.

## 2 Materials and methods

### 2.1 Registration

Our study registered at Research Registry by this identifying number UIN researchregistry8258.

### 2.2 Ethics

The institutional review board approved the study (H-01-R-012, IRB log No.21-228).

### 2.3 Study design

The study design was a retrospective cross-sectional and the work has been reported in line with the STROCSS criteria [17].

### 2.4 Setting and time frame of research

The study was conducted at King Fahad Medical City-Riyadh between January 2010 to December 2019.

### 2.5 Data collection

Patient data, including clinical notes, demographics, and MRI studies, were retrieved from patient medical records and the standard picture archiving and communication system (PACS). The inclusion criteria included patients with FT-RCT of the superior rotator cuff tendons (supraspinatus or infraspinatus) who had shoulder MRI scans from January 2010 to December 2019. The exclusion criteria included prior history of trauma, shoulder surgery, or documented prior repetitive shoulder injections.

All patients were scanned using two MRI systems with different magnet strengths (GE 1.5 T and Siemens 3 T). The MRI scan protocols were Proton density with fat saturation (PD fat sat) in the coronal plane, Proton density with fat saturation in the axial plane, T2 fat sat in the sagittal plane, and T1W1 in the sagittal plane. Patients were scanned in the supine position using a dedicated shoulder coil.

The studies were reviewed retrospectively by one MSK radiologist with more than eight years of experience in the subspecialty of MSK Radiology. The collected data and variables were FT-RCT cases, the tear size, muscle fatty degeneration, deltoid tear, and deltoid tear location, whether it involves the anterior, middle, or posterior portion. FT-RCT size grading system was defined as small (<1 cm), medium (1–3 cm), large (3–5 cm), and massive (>5 cm). RCT muscle fatty degeneration was graded using Goutallier's classification system (0 for normal muscle, 1 for some fatty streaks, 2 for less than 50% fatty muscle atrophy, 3 for 50% fatty muscle atrophy, and 4 for more than 50% muscle atrophy). Moreover, AH interval was recorded and analyzed on the anterior-posterior projection of shoulder radiography for those patients with FT-RCT.

### 2.6 Statistical analysis

All data were entered and analyzed using SPSS Statistics for Windows, version 25 (IBM Corp., Armonk, N.Y., USA). Tests of normality for continuous primary continuous variables showed that the data was skewed and did not follow a normal distribution. Hence median and interquartile (IQR) was used to represent continuous variables. Mann-Whitney *U* test and Kruskal-Wallis H test compared continuous variables with RCT. Pearson's Chi-square test was used to find a relationship between RCT and other categorical variables. Categorical variables such as gender, side, X-ray, and RCT muscle fatty degeneration were expressed as numbers and percentages. The correlation between deltoid tears and RCT size was tested. In addition, AH interval measurements were compared between the deltoid and non-deltoid tear. A p-value of <0.05 indicated a statistically significant difference.

## 3 **Results**

### 3.1 Participants

A total of two thousand one hundred and twenty-three (n = 2123) MRI shoulder scans were collected during the period between January 2010 and December 2019. After excluding cases without FT-RCT, the remaining cases were 271 cases, 160 of the right shoulder and 111 of the left shoulder. The number of male patients was 100, while female patients were 171.

### 3.2 Outcomes

The average age for patients with FT-RCT in our study was 65 years. Most patients had isolated FT-RCT of the supraspinatus tendon (n = 215, 79.3%). Both supraspinatus and tendon had a complete full-thickness tear in 53 patients (19.6%). Only three patients (1.1%) had isolated infraspinatus tendon tears. The degree of muscle fatty degeneration was 0 in 28 patients (10.3%), 1 in 107 patients (39.5%), 2 in 76 patients (28%), 3 in 40 patients (14.8%), and 4 in 20 patients (7.4%), Table 1.

**Table 1:** Demographic and clinical characteristics of patients (n = 271).

Variables	Description	n	%
Gender	Female	171	63.1
	Male	100	36.9
Side	Left	111	41.0
	Right	160	59.0
X-ray	Yes	158	58.3
	No	113	41.7
RCT disease (SST/IST)	IST	3	1.1
	SST	215	79.3
	Both	53	19.6
RCT muscle fatty degeneration	0	28	10.3
	1	107	39.5
	2	76	28.0
	3	40	14.8
	4	20	7.4
Deltoid and Non-Deltoid	Deltoid	19	7.0
	Non-Deltoid	252	90.4

RCT - Rotator Cuff Tear, SST - supraspinatus tendon, IST - infraspinatus tendon.

Our study found that 19 cases (7%) of a deltoid tear were encountered in patients with FT-RCT. Fifteen cases were on the right side (9.4%), and only four were on the left side (3.6%). Statistically, Man-Whitney U indicated a significant association between deltoid tears and FT-RCT, P < 0.05, Table 2.

**Table 2:** Impact and association between deltoid tear and clinical parameters of the study.

Variables	Description	Deltoid	Non-Deltoid	P - value
Age (years)	Median (IQR)	64 (13)	59 (13)	*0.003
Gender	Female	11 (6.4%)	160 (93.6%)	0.626
	Male	8 (8%)	92 (92%)	
Side	Left	4 (3.6%)	107 (96.4%)	0.067
	Right	15 (9.4%)	145 (90.6%)	
	No	10 (8.8%)	103 (91.2%)	
RCT disease (SST/IST)	IST	0 (0%)	3 (100%)	*<0.001
	SST	2 (0.9%)	213 (99.1%)	
	Both	17 (32.1%)	36 (67.9%)	
RCT Muscle Fatty Degeneration	0	0 (0%)	28 (100%)	*<0.001
	1	1 (0.9%)	106 (99.1%)	
	2	3 (3.9%)	73 (96.1%)	
	3	4 (10%)	36 (90%)	
	4	11 (55%)	9 (45%)	
AH interval mm	Median (IQR)	3 (6)	5 (3)	*0.045

RCT - Rotator Cuff Tear, SST - supraspinatus tendon, IST - infraspinatus tendon.

*p-value <0.05.

**Fig. 1:**
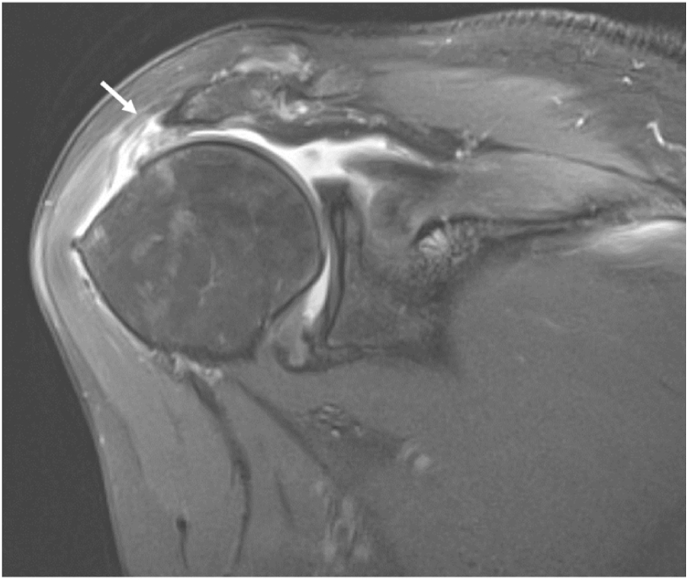
Coronal proton density fat-saturation MR image exhibits partial undersurface tear of the middle portion of the deltoid muscle (white arrow) at its acromial insertion, in association with a massive retracted full-thickness tear of the supraspinatus tendon. Note the proximal migration of the humeral head and the reduced acromio-humeral interval.

Eleven females (6.4%) and 8 males (8%) with deltoid tears. Tears of the deltoid were predominantly seen in the middle segment (Fig. 1). One patient had edema in the posterior segment alone. Table 1.

**Fig. 2:**
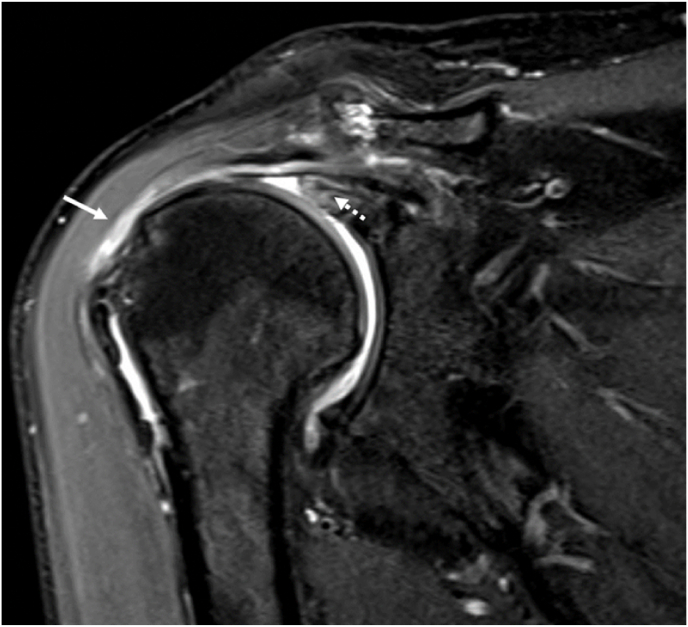
Coronal proton density fat-saturation MR image demonstrates partial under surface tear and fibers fraying at the myotendinous junction of the middle portion of the deltoid muscle (white arrow), which is in close proximity to the humeral greater tuberosity, in another patient presented with a chronic moderate retracted full-thickness tear of the supraspinatus tendon (dotted white arrow).

One deltoid tear was associated with moderate RCT size. Seven (16.7%) deltoid tears were associated with large RCT and eleven (42.3%) with massive RCT (Fig. 2). Our analysis suggested a significant correlation between the deltoid tear and the RCT size p < 0.05, Table 3.

**Table 3:** The correlation between the deltoid and non-deltoid cases and the size of RCT.

Variables			Deltoid Abnormality		Total	P value
			Deltoid	Non-Deltoid		
RCT size	Small	N	0	76	76	<0.001
		%	0.0%	100.0%	100.0%	
	Moderate	N	1	126	127	
		%	0.8%	99.2%	100.0%	
	Large	N	7	35	42	
		%	16.7%	83.3%	100.0%	
	Massive	N	11	15	26	
		%	42.3%	57.7%	100.0%	

We found that 15 out of 19 patients had a background of grade 3 or 4 (10%,55%, respectively) rotator cuff muscle fatty degeneration. On the other hand, only 45 patients without deltoid tears had high grades 3 or 4 (90%, 45%, respectively) muscle fatty degeneration. The average AH interval measurement for patients with FT-RCT was 5 mm. In contrast, the average measurement of AH interval for patients associated with a deltoid tear was 3 mm, a statistically significant p-value of 0.045, Table 2.

## 4 Discussion

Deltoid tears are not commonly seen in daily clinical practice. However, there are well-established risk factors related to deltoid tears, including a history of trauma, surgical intervention, and shoulder injections. Deltoid detachment following surgery is a well-recognized complication, especially with acromioplasty [12]. This study aimed to investigate the incidence and the association of deltoid tear with FT-RCT in patients with no previous shoulder trauma or surgery besides evaluating the association of deltoid tear with different grades of FT-RCT.

This study revealed that the association of deltoid tear with large and massive FT-RCT is considered significant, with an incidence rate of 7%. Deltoid tears associated with massive RCT have been increasingly reported in the literature through several small case series reports [[18], [19], [20], [21], [22], [23], [24], [25]]. In one large sample study, Ilaslan et al. reported 24 deltoid tears among 8,562 shoulders MRI studies with a prevalence of 0.3%. [15]. All these tears were associated with FT-RCT. Lecours et al. reviewed 380 shoulder MRI studies, and they reported a 9.2% prevalence of deltoid abnormalities without a clear description of the incidence of the deltoid tear and RCT grads [26]. The true incidence of deltoid tears with FT-RCT is underreported in the literature. Lecours et al. study has no details of the association as it was an abstract conference in addition to the retrospective nature and recruiting minimal abnormalities of deltoid tears despite increasing incidence [26]. It is important to emphasize the descriptive imaging characteristics of the deltoid tears and their association with the grading of FT-RCT in patients without previous RCT trauma or surgery.

The average age for patients with deltoid tears was 65 years, as reported in previous studies [11]. In this study, the deltoid tears were higher in female patients >60. This could be explained by the expected prevalence of lower muscle mass in those patients and the increased occurrence of degenerative RCT [14,15]. A recent study indicates that deltoid tears are more common among men patients who practice contact sports and vigorous physical activity [16].

This study indicates that pain and reduced range of abduction was the most common presenting symptom in the study group. Also, most of our patients had isolated supraspinatus tendon full-thickness tears. Complete full-thickness tear of the supraspinatus tendon was infrequently observed. Many patients had long-standing rotator cuff degenerative tear features, including muscle fatty degeneration and decreased AH interval.

In the current study, the deltoid tear predominantly involved the middle portion in all cases, which concord with the postulated injury mechanism observed in previous studies [[14], [15], [16]]. Few theories elucidate the possible pathogenesis and mechanism of a deltoid muscle tear in patients with massive RCT. One of the possible explanations is that following massive rotator cuff tears; patients develop superior subluxation of the humeral head. This, in effect, can lead to the humeral head rubbing against the overlying deltoid muscle near the enthesis. This theory is supported by evidence that most tears involve the deltoid muscle under-surface [14]. Muscle fiber edema was noted in ten additional patients, and it involved the middle segment except in one case where the edematous changes were solely noted in the posterior segment.

Furthermore, this study found a strong correlation between large and massive RCT with a deltoid tear. In previous studies in which, 58.3% of patients with deltoid tears had massive rotator cuff tears [15,16]. A previous study showed that most patients with deltoid abnormalities associated with rotator cuff tears had developed moderate to severe fatty degeneration of the rotator cuff muscles [15,16]. A similar observation was noted in which 65% of patients had developed a background of grade 3 or 4 rotator cuff muscle fatty degeneration. The AH interval was significantly reduced in patients with deltoid tears, with an average of 3 mm compared to 5 mm in the non-deltoid tear group. Moreover, the most common location of tears is at the myotendinous junction, which is the area most susceptible to friction by the greater tuberosity of the humerus. It was found that most patients with deltoid tears associated with FT-RCT had high riding humeral heads.

This study findings would significantly contribute to the literature regarding the incidence and association of deltoid tears, especially the size of RCT. However, study limitations such as the smaller recruiting population, retrospective study design, and recall bias are unavoidable. In addition, all cases were observed and reported by one radiologist, which may impact the overall incidence.

## 5 Conclusion

Deltoid tears are infrequently seen with FT-RCT, especially in patients without prior surgical or interventional repairs. However, this study indicated a strong association between deltoid tears and the size of rotator cuff tears and tendon retraction, and the incidence reached almost 7% of the recruited cases and the middle segment of the deltoid muscle was the most common location of the tears.

## Ethical approval

The study was gathered at King Fahad Medical City (KFMC), Riyadh, Saudi Arabia. The institutional review board at KFMC approved the study (KACST, KSA: H-01-R-012, IRB log No.21-228).

## Sources of funding

None.

## Author contribution

Abdulrahman Alharbi and Salem Bauones designed the study; AA collected the data; Salem Bauones and Mamdouh Almalki read the images; Abdulrahman M. Alfuraih and Mohammed Alsaadi processed the data and the statistical analyses; Abdulrahman Alharbi, Mamdouh Almalki and Salem Bauones were involved in data interpretation; Abdulrahman Alharbi, Salem Bauones and Mohammed Alsaadi prepared the manuscript; Salem Bauones and Mohammed Alsaadi and Abdulrahman M. Alfuraih were contributed to reviewing and revising the manuscript and approved the final draft.

## Trial registeration number

1. Name of the registry: research registry
2. Unique Identifying number or registration ID: researchregistry8258
3. Hyperlink to your specific registration (must be publicly accessible and will be checked): researchregistry8258

## Guarantor

Corresponding author Mohammed Alsaadi,

Radiology and Medical Imaging Department, College of Applied Medical Sciences in Al-Kharj, Prince Sattam Bin Abdulaziz University, Al-Kharj 11942, Saudi Arabia.

Office 145, PO Box 422, Al-Kharj 11942. Email: m.alsaadi@psau.edu.sa.

## Consent

Written informed consent was obtained from the patient for publication of this study and accompanying images.

## Disclosure

The authors have no conflicts of interest to disclose.

## Provenance and peer review

Not commissioned, externally peer-reviewed.

## Declaration of competing interest

None.
